# Application of Proteomics for the Investigation of the Effect of Initial pH on Pathogenic Mechanisms of *Fusarium proliferatum* on Banana Fruit

**DOI:** 10.3389/fmicb.2017.02327

**Published:** 2017-11-29

**Authors:** Taotao Li, Qixian Wu, Yong Wang, Afiya John, Hongxia Qu, Liang Gong, Xuewu Duan, Hong Zhu, Ze Yun, Yueming Jiang

**Affiliations:** ^1^Key Laboratory of Plant Resource Conservation and Sustainable Utilization, South China Botanical Garden, Chinese Academy of Sciences, Guangzhou, China; ^2^College of Life Science, University of Chinese Academy of Sciences, Beijing, China; ^3^Zhong Shan Entry-Exit Inspection and Quarantine Bureau, Zhong Shan, China

**Keywords:** environmental pH-value, *Fusarium proliferatum*, secretome, cell wall degrading enzymes, oxidation-reduction process

## Abstract

*Fusarium proliferatum* is an important pathogen and causes a great economic loss to fruit industry. Environmental pH-value plays a regulatory role in fungi pathogenicity, however, the mechanism needs further exploration. In this study, *F. proliferatum* was cultured under two initial pH conditions of 5 and 10. No obvious difference was observed in the growth rate of *F. proliferatum* between two pH-values. *F. proliferatum* cultured under both pH conditions infected banana fruit successfully, and smaller lesion diameter was presented on banana fruit inoculated with pH 10-cultured fungi. Proteomic approach based on two-dimensional electrophoresis (2-DE) was used to investigate the changes in secretome of this fungus between pH 5 and 10. A total of 39 differential spots were identified using matrix-assisted laser desorption/ionization tandem time-of-flight mass spectrometry (MALDI-TOF/TOF-MS) and liquid chromatography electrospray ionization tandem mass spectrometry (LC-ESI-MS/MS). Compared to pH 5 condition, proteins related to cell wall degrading enzymes (CWDEs) and proteolysis were significantly down-regulated at pH 10, while proteins related to oxidation-reduction process and transport were significantly up-regulated under pH 10 condition. Our results suggested that the downregulation of CWDEs and other virulence proteins in the pH 10-cultured *F. proliferatum* severely decreased its pathogenicity, compared to pH 5-cultured fungi. However, the alkaline environment did not cause a complete loss of the pathogenic ability of *F. proliferatum*, probably due to the upregulation of the oxidation-reduction related proteins at pH 10, which may partially compensate its pathogenic ability.

## Introduction

*Fusarium proliferatum* is a polyphagous fungus with a broad host range and is often isolated from several agriculturally important crops, including wheat (Palacios et al., 2015), banana (Li et al., 2012), citrus (Amby et al., 2015), etc. Various mycotoxins produced by *F. proliferatum* are harmful to human and animal health. Therefore, controlling its infection is important for food safety. Ambient pH is an important environmental factor, which could influence the survival, proliferation, and pathogenicity of microorganism. Weak alkaline environment can significantly inhibit the growth of fungi and their infection to plants (Prusky and Yakoby, 2003). Meanwhile, the ambient pH has the critical role in determining the transcriptional levels of many genes, affecting growth, physiology, and differentiation processes (Lamb et al., 2001). Pathogens also boost some proteins to resist harsh environment and increase adaptability (Bi et al., 2016). Numerous researches were conducted to investigate the pH signal transduction and relationship between the pH regulation and fungal pathogenicity (Penalva et al., 2008). Transcription factor *PacC* appears to be necessary for the appropriate regulation of physiological processes *Sclerotinia sclerotiorum* (Rollins, 2003). *Pac1* is reported to regulate *Tri* gene expression and trichothecene production in *Fusarium graminearum* (Merhej et al., 2011). However, the regulatory mechanism is still not clearly understood, and little information was available on the effect of pH on the secretome of *Fusarium*.

Some pathogenic microorganisms usually secret proteins to facilitate their infection and host colonization (Zhang et al., 2014). Fruit pathogens can contribute to the acidification or alkalinization of the host environment, and the capability has been used to divide fungal pathogens into acidifying and/or alkalinizing classes (Bi et al., 2016). To comprehensively unravel how pathogens manipulate the infection process, the investigation of secretome changes under different ambient pH conditions will be useful to explore the pathogenic mechanism of fungal pathogens. The secreted pathogenicity factors are well known for their ability to help the pathogen successfully colonize and invade the targeted host (Zong et al., 2015). In recent years, proteomic approach has been widely used to explore the secretome change and infection mechanism of several fungal pathogens (Li et al., 2012; Meijer et al., 2014; Lakshman et al., 2016). Additionally, proteomics analyses were used to comprehensively characterize infection-specific protein expression pattern of early defense-related signaling in *Medicago truncatula* (Trapphoff et al., 2009). However, little information is available for *F. proliferatum*, especially for the secretome. Therefore, understanding secretomics can provide vital information for advances in the identification of extracellular proteins with a potential role in pathogenicity of *F. proliferatum*.

In previous research of our lab, we investigated the effect of different initial pH values (ranging from 3 to10) on the growth of *F. proliferatum*, and the results showed that pH 5 and 10 had no influence on the growth but affected fusaic acid (FA) production (Li et al., 2012). Additionally, our previous research also showed that the initial pH 5 and 10 showed no significant effect on the growth rate of *F. proliferatum* (Li et al., 2017). Based on these results, the effect of pH 5 and 10 on the pathogenic ability of *F. proliferatum* needs further analysis, especially at secreted proteins. In the present study, the effect of initial pH values on the growth of *F. proliferatum* was further verified on PDA plate culture media, and inoculation experiment on banana fruit was performed to verify the effect of these two pH values on the pathogenicity of *F. proliferatum*. Additionally, the secretome change was comparatively analyzed at pH 5 and 10, using a gel based proteomic approach. The objective of this study was to investigate the molecular mechanism of different starting environmental pH values in regulating the pathogenicity of *F. proliferatum*. This study will be helpful in providing insightful knowledge of the pathogenic mechanism of *F. proliferatum*, which will also facilitate the development of new antifungals/fungicides.

## Materials and methods

### Fungal strains and growth conditions

*Fusarium proliferatum* was isolated from carambola and then stored in 50% glycerol at −80°C. *Fusarium proliferatum* was grown for 7 days at 28°C on PDA (Oxoid, Basingstoke, Hampshire, England) plates. Then six small plates (5 mm) was cut and transferred to Czapek's broth medium (CB) modified with NaOH or HCl to maintain their starting pH with the range of 5.0 ± 0.2 and 10.0 ± 0.2, respectively. The pH value was measured by Ultrabasic pH Meters, UB-7 (Denver Instrument, Arvada, USA). The conical flask containing 150 mL above cultures was incubated at 28°C for 10 days in an orbital shaker (200 rpm). Mycelium and medium were separated by filtering with a vacuum pump. Three independent biological replicates were conducted.

### Ripe banana inoculation with *F. proliferatum*

Yellow ripe banana (*Musa acuminate* L. AAA group, cv. Brazilian) fruit were bought from a commercial orchard in Guangzhou, China. Fruit fingers with uniform shape, color, and size were selected for further experiment. The culture of *F. proliferatum* under different initial pH conditions was filtered using two layers of gauze, and the spore solution was diluted to 1 × 10^6^ spores/mL. Fruit fingers were washed by sterile water, then wounded with a nail (1 mm wide, 2 mm deep) and inoculated with 15 μL aqueous conidia suspension. The inoculated fruits were stored at 22°C and 85% relative humidity for 3 days. Three biological replicates with 12 fruit fingers for each were conducted.

### Secreted protein isolation, two-dimensional gel electrophoresis, in-gel digestion, mass spectrometry (MS), and database searching

*Fusarium proliferatum* was cultured in CB and used for secreted proteins extraction. After removing the residual mycelia and other debris from the media by filtrating, the secreted proteins were isolated from the filtrate according to the methods described by Li et al. (2016). The protein concentration was measured using the Bio-Rad protein assay kit (Bio-Rad, USA). Two-dimensional electrophoresis (2-DE) was performed using 2 mg of protein sample to rehydrate gel strips (IPG strip, pH 4–7, 17 cm; Bio-Rad, USA). After stained with Coomassie Brilliant Blue R-250, PDQuest™ Version 8.0.1 (Bio-Rad) was used to analyze the gel images. At least three independent biological replicate gels were run. Spots with more than a three-fold differential accumulation in three independent gels (*p* < 0.05) were excised and then used for protein identification.

In-gel tryptic digestion and MALDI-TOF/TOF analysis were performed according to mature research method (Li et al., 2015). Mascot software 2.3.02 (Matrix Science, London, UK) was used for database sequence searches against UniProt_Fusarium database (http://www.uniprot.org/uniprot/?query=fusarium&offset=50&sort=score&columns=id%2centry+name%2creviewed%2cprotein+names%2cgenes%2corganism%2clength) with 292990 sequences. Protein candidates provided by the combined PMF and MS/MS search were considered as valid when the global Mascot score was greater than Significance Score (58) with a significance level of *e*-value < 0.05. For LC-ESI-MS/MS analysis, the method described by Li et al. (2016) was carried out. The same software and database described above were used for protein identification. To reduce the probability of false peptide identification, peptides with ion scores greater than “identity” score were counted as identified. Each reliably identified protein contained at least one unique peptide.

### Prediction of extracellular location of identified proteins

Classical secreted proteins were identified by SignalP 4.0 (http://www.cbs.dtu.dk/services/SignalP/) and non-classical protein secretion was analyzed by SecretomeP 1.0b (http://www.cbs.dtu.dk/services/SecretomeP/).

### Quantitative real-time PCR validation

The mycelia of *F. proliferatum* cultured under different pH values for 10 days was used for RNA extraction. The total RNA extraction and qRT-PCR were conducted according to our previous methods (Li et al., 2017). The specific primers used for qRT-PCR analysis were shown in Supplementary Table S1. Three independent biological replicates were conducted.

## Results

### Infection ability of *F. proliferatum* under acidic or alkaline environment

**Figure 1:**
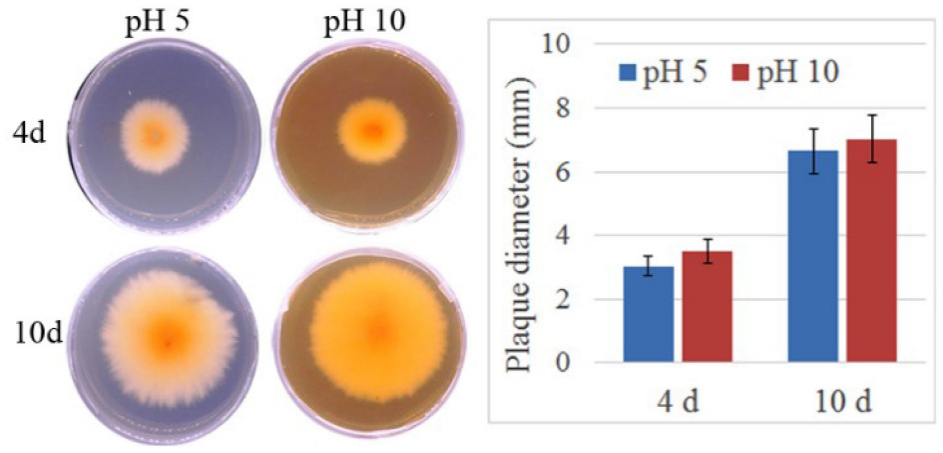
The area of mycelium under differential pH values. *Fusarium proliferatum* was cultured for 7 days at 28°C on PDA plates, six small plates (5 mm) was cut and transferred to Czapek's broth medium (CB) modified with NaOH or HCl to maintain their starting pH with the range of 5.0 ± 0.2 and 10.0 ± 0.2, respectively. After cultured 10 days, the area of mycelium was calculated.

**Figure 2:**
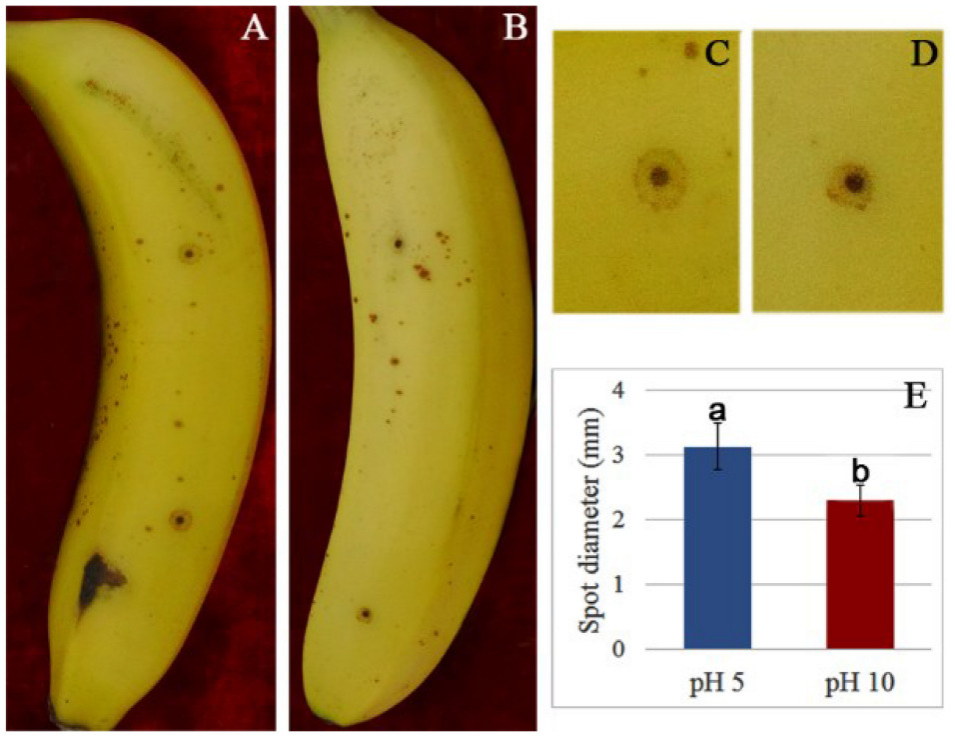
The disease spot of banana fruit after inoculated with *F. proliferatum*. **(A,C)** Banana fruit infected by *F. proliferatum* cultured at pH 5. **(B,D)** Banana fruit infected by *F. proliferatum* cultured at pH 10. **(E)** Spot diameter of banana fruits inoculated with *F. proliferatum*. *F. proliferatum* under different initial pH conditions was filtered using two layers of gauze, and the spore solution was diluted to 1 × 10^6^ spores/mL. Banana fruits were wounded with a nail (1 mm wide and 2 mm deep) and inoculated with 15 μL aqueous conidia suspension.

To verify the effect of pH value on the fungus growth rate, *F. proliferatum* was cultured on PDA plates under pH 10 for 10 days; pH 5 set as control. No significant difference of growth rate was shown in the two different pH values (Figure 1). After inoculated to the ripen banana peel, a smaller lesion diameter was found on the ripe banana peel inoculated with pH 10-cultured *F. proliferatum* than that with pH 5 (Figure 2). It seemed that weak alkaline environment decreased the pathogenicity slightly, small difference between pH 10 and pH 5 was observed in the pathogenicity of *F. proliferatum*.

### 2-DE and protein identification

**Figure 3:**
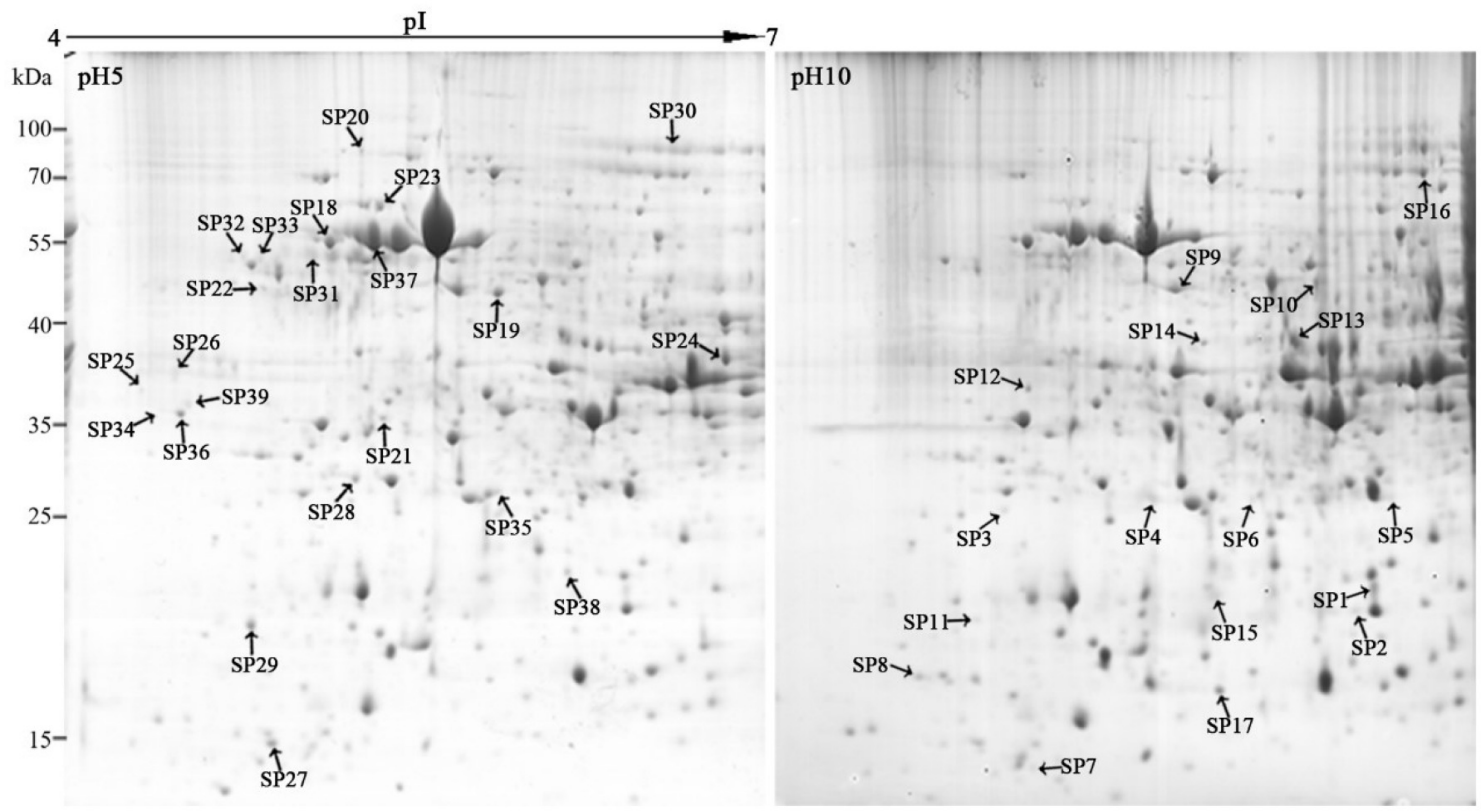
Representative two-dimensional electrophoresis maps of *Fusarium proliferatum*. Total secreted proteins were extracted from *F. proliferatum* after 10 d culture under pH 5 or pH 10 condition. The location of differentially expressed proteins were identified successfully.

Two milligrams of total proteins were separated using 2-DE, and more than 600 protein spots were detected in each gel (Figure 3). After comparative analysis, protein spots showing statistically significant (*p* < 0.05) changes and more than three-fold in relative abundance between pH 5 and 10 were selected for identification. Due to the lack of *F. proliferatum* genome information, only 17 protein spots were successfully identified by means of matrix-assisted laser desorption/ionization-time of flight mass spectrometry (MALDI-TOF MS). The unidentified protein spots were then analyzed using liquid chromatography-electronic spray ionization-tandem mass spectrometry (LC–ESI–MS/MS), 22 of them were successfully identified via searching NCBI nr database (Figure 3).

**Figure 4:**
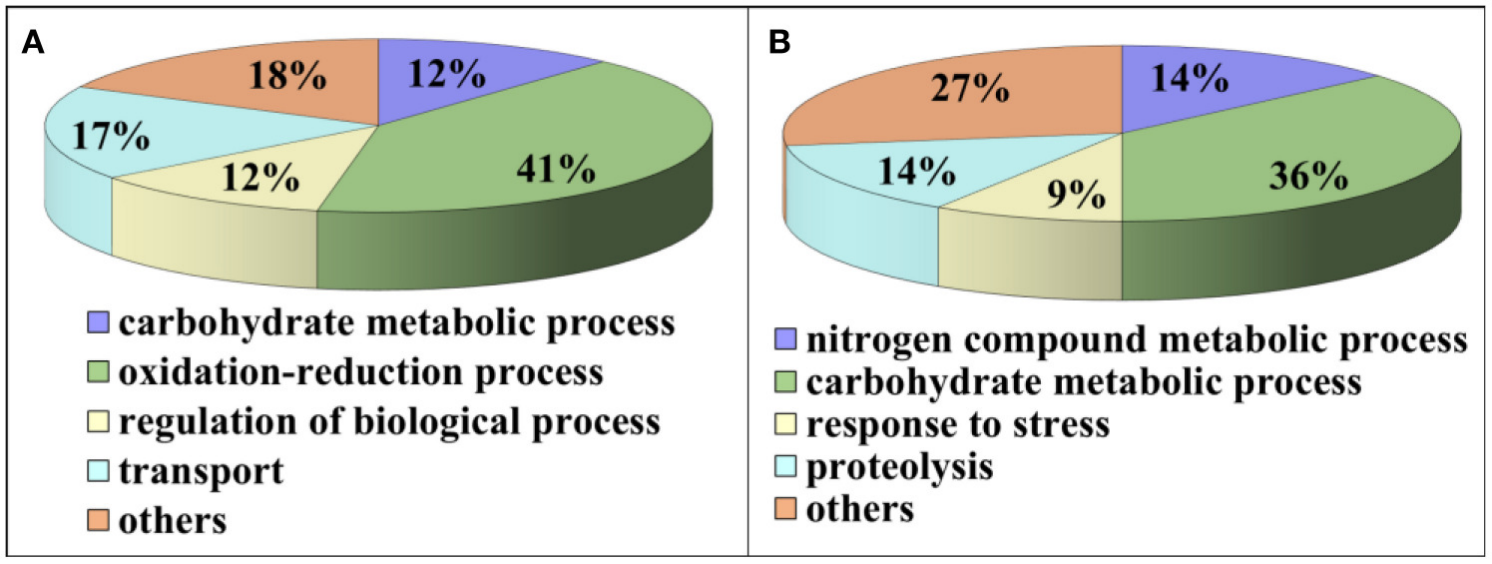
Functional classification of differential expressed proteins. Proteins were classified using Blast2Go base on biological process. **(A)** Up-regulated proteins at pH 10 compared to pH 5. **(B)** Down-regulated proteins at pH 10 compared to pH 5.

Compared to pH 5, 17 proteins were up-regulated at pH 10 (Table 1), and 22 protein spots were down-regulated (Table 2). The up-regulated proteins were also classified into five clusters using Blast2GO base on biological process, including carbohydrate metabolism (2 spots), oxidation-reduction process (7 spots), transport (3 spots), regulation of biological process (2 spots), and unknown function (3 spots) (Figure 4A, Table 1). Meanwhile, the down-regulated proteins were categorized into five groups, including carbohydrate metabolism (8 spots), nitrogen compound metabolic process (3 spots), proteolysis (3 spots), response to stress (2 spots), and unknown function (6 spots) (Figure 4B, Table 2). All the spots with unknown function were also searched against PROSITE database for more protein domain information and only spot SP25 was hit by acyl carrier protein phosphopantetheine domain.

**Table 1:** Up-regulated secretory proteins in *F. proliferatum* under alkaline environment.

**Sample name**	**SignalP**	**Changefold**	**Protein ID**	**Protein description**	**Theo Mr/PI**	**2D Mr/PI**	**Protein score**
**OXIDATION-REDUCTION PROCESS (7)**							
SP1	P	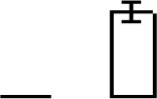	tr|S0E3Q1|S0E3Q1_GIBF5	Related to thioredoxin reductase	34.39/5.9	21.55/4.46	104.22
SP2	P	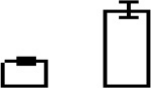	tr|S0E3Q1|S0E3Q1_GIBF5	Related to thioredoxin reductase	34.39/5.9	20.62/6.41	50.34
SP3	P	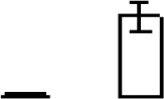	tr|S0EF18|S0EF18_GIBF5	Superoxide dismutase	25.07/8.03	26.25/5.06	45.91
SP4	+	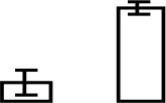	tr|S0EEC6|S0EEC6_GIBF5	Superoxide dismutase	28.38/6.23	29.08/5.68	221.98
SP5	+	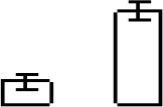	tr|S0EEC6|S0EEC6_GIBF5	Superoxide dismutase	28.38/6.23	26.51/6.53	115.28
SP6	+	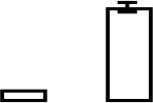	tr|S0EEC6|S0EEC6_GIBF5	Superoxide dismutase	28.38/6.23	26.56/6.0	518.16
SP7	P	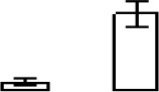	tr|S0EMH8|S0EMH8_GIBF5	Related to tyrosinase (Monophenol monooxygenase)	76.19/5.97	14.41/5.17	32.24
**CARBOHYDRATE METABOLIC PROCESS (2)**							
SP8	+	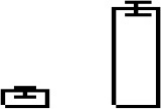	tr|S0EJD2|S0EJD2_GIBF5	Related to covalently-linked cell wall protein	41.90/4.58	17.81/4.75	117.73
SP9	+	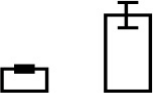	tr|S0E9I4|S0E9I4_GIBF5	Related to SPR1-exo-1 3-beta-glucanase	49.00/6.19	45.73/5.79	115.39
**REGULATION OF BIOLOGICAL PROCESS (2)**							
SP10	–	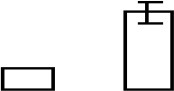	tr|A0A071M2G2|A0A071M2G2_9BURK	Elongation factor	41.76/4.92	48.74/6.3	765.52
SP11	–	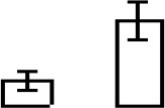	tr|W7MJH9|W7MJH9_GIBM7	30S ribosomal protein S13	13.32/12.08	20.56/4.96	71.9
**TRANSPORT (3)**							
SP12	+	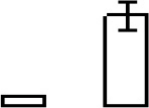	tr|A0A071M865|A0A071M865_9BURK	Porin	39.18/9.41	34.13/5.16	70.34
**OTHERS (3)**							
SP13	+	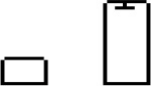	tr|A0A071MG81|A0A071MG81_9BURK	Iron ABC transporter substrate-binding protein	39.87/9.7	39.58/6.21	72.02
SP14	+	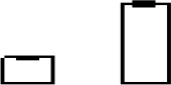	tr|A0A071MG81|A0A071MG81_9BURK	Iron ABC transporter substrate-binding protein	39.87/9.7	39.12/5.86	42.42
SP15	+	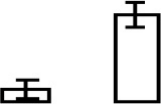	tr|S0EHG0|S0EHG0_GIBF5	Uncharacterized protein	21.39/5.92	21.81/5.89	39.9
SP16	+	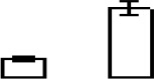	tr|C7Z0J4|C7Z0J4_NECH7	Predicted protein	12.81/4.33	74.06/6.73	67.3
SP17	+	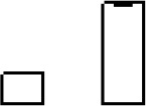	tr|D2IKP5|D2IKP5_9HYPO	APS1	571.27/5.9	17.08/5.89	72.2

*A total of 17 proteins were identified and up-regulated at pH 10 compared to pH 5. Protein accumulation is represented by the column configuration, and accumulation of pH 5 and pH 10 was shown from left to right. +, Proteins with positive result from SignalP; P, Proteins with positive result from SecretomeP; –: Proteins with negative result from both SignalP and SecretomeP. Theo Mr/PI: Theoretical molecular mass and isoelectric point based on amino acid sequence of the identified proteins. 2D Mr/PI: Experimental molecular mass and isoelectric point estimated from the 2D gels. The error bar represents the standard error of the average over three independent biological replicate gels. The same is for Table 2*.

**Table 2:** Down-regulated secretory proteins in *F. proliferatum* under alkaline environment.

**Sample name**	**SignalP**	**Changefold**	**Protein ID**	**Protein description**	**Theo Mr/PI**	**2D Mr/PI**	**Protein score**
**CARBOHYDRATE METABOLIC PROCESS (8)**							
SP18	+	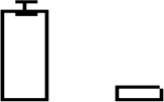	tr|S0E1L4|S0E1L4_GIBF5	1 3-beta-glucanosyltransferase	48.81/4.71	56.58/5.19	88.74
SP19	+	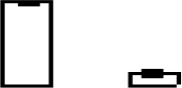	tr|S0E9I4|S0E9I4_GIBF5	Related to SPR1-exo-1,3-beta-glucanase	49.00/6.19	45.94/5.92	274
SP20	+	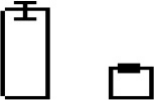	tr|S0DSA1|S0DSA1_GIBF5	Related to glucan 1 3-beta-glucosidase	42.49/4.51	80/5.3	41.65
SP21	P	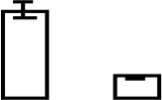	tr|S0E8F8|S0E8F8_GIBF5	Related to endo alpha-1 4 polygalactosaminidase precusor	38.24/9.6	32.21/5.41	104.13
SP22	–	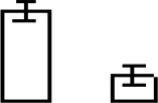	tr|A0A063XCK4|A0A063XCK4_BACIU	Enolase	46.61/4.4	48.07/4.92	84.67
SP23	+	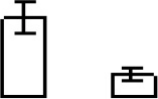	tr|X0HDL4|X0HDL4_FUSOX	Gluconolactonase	43.25/5.18	64.84/5.39	144.47
SP24	+	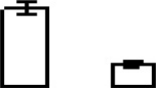	tr|S0DM93|S0DM93_GIBF5	Probable cellulase	37.12/7.71	38/6.9	23.84
SP25	+	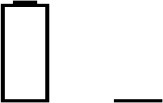	tr|S0DSR5|S0DSR5_GIBF5	Probable rAsp f 9 allergen	29.51/4.26	34.05/4.34	67.2
**NITROGEN COMPOUND METABOLIC PROCESS (3)**							
SP26	P	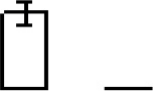	tr|X0J1C7|X0J1C7_FUSOX	Adenosinetriphosphatase	190.16/4.99	35.00/4.50	67.2
SP27	P	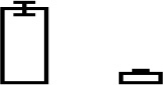	tr|Q05GS8|Q05GS8_GIBFU	Peptidyl-prolyl cis-trans isomerase	12.05/4.59	15.04/4.98	337
SP28	P	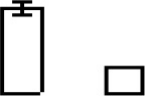	tr|X0CL22|X0CL22_FUSOX	Serine/threonine protein kinase	50.9/7.29	27.6/5.22	24.52
**RESPONSE TO STRESS (2)**							
SP29	P	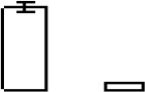	tr|S0E8X5|S0E8X5_GIBF5	Probable peroxisomal membrane protein	18.14/4.96	19.84/4.88	154
SP30	+	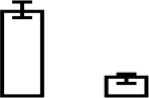	tr|W9ZB77|W9ZB77_FUSOX	Catalase-peroxidase	85.26/7.04	81.03/6.70	381
**PROTEOLYSIS (3)**							
SP31	+	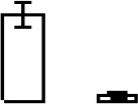	tr|N1RNP5|N1RNP5_FUSC4	Antigen	31.81/5.18	54.78/5.07	109
SP32	+	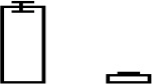	tr|S0DTT6|S0DTT6_GIBF5	Probable PRC1-carboxypeptidase y, serine-type protease	60.95/4.93	54.08/4.81	116
SP33	+	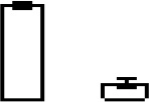	tr|S0E6V7|S0E6V7_GIBF5	Related to aspartic proteinase	41.80/4.4	53.65/4.89	65.67
**OTHERS (6)**							
SP34	+	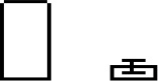	tr|C7ZIQ5|C7ZIQ5_NECH7	Predicted protein	27.52/4.79	32.59/4.44	118
SP35	P	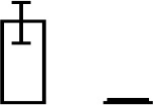	tr|A0A016PIY6|A0A016PIY6_GIBZA	Fusarium graminearum chromosome 1	60.95/10.23	26.79/5.91	72.1
SP36	+	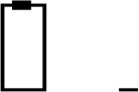	tr|S0EL20|S0EL20_GIBF5	Uncharacterized protein	27.83/4.48	32.39/4.52	278
SP37	+	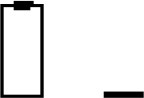	tr|S0EAM0|S0EAM0_GIBF5	Uncharacterized protein	18.11/9.69	57.88/5.34	88.7
SP38	+	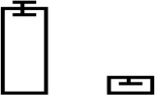	tr|W7MXE0|W7MXE0_GIBM7	Uncharacterized protein	23.82/7.36	22.39/6.23	154
SP39	+	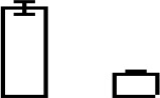	tr|S0EL20|S0EL20_GIBF5	Uncharacterized protein	27.83/4.48	32.80/4.57	83.5

### Up-regulated proteins at pH 10

**Figure 5:**
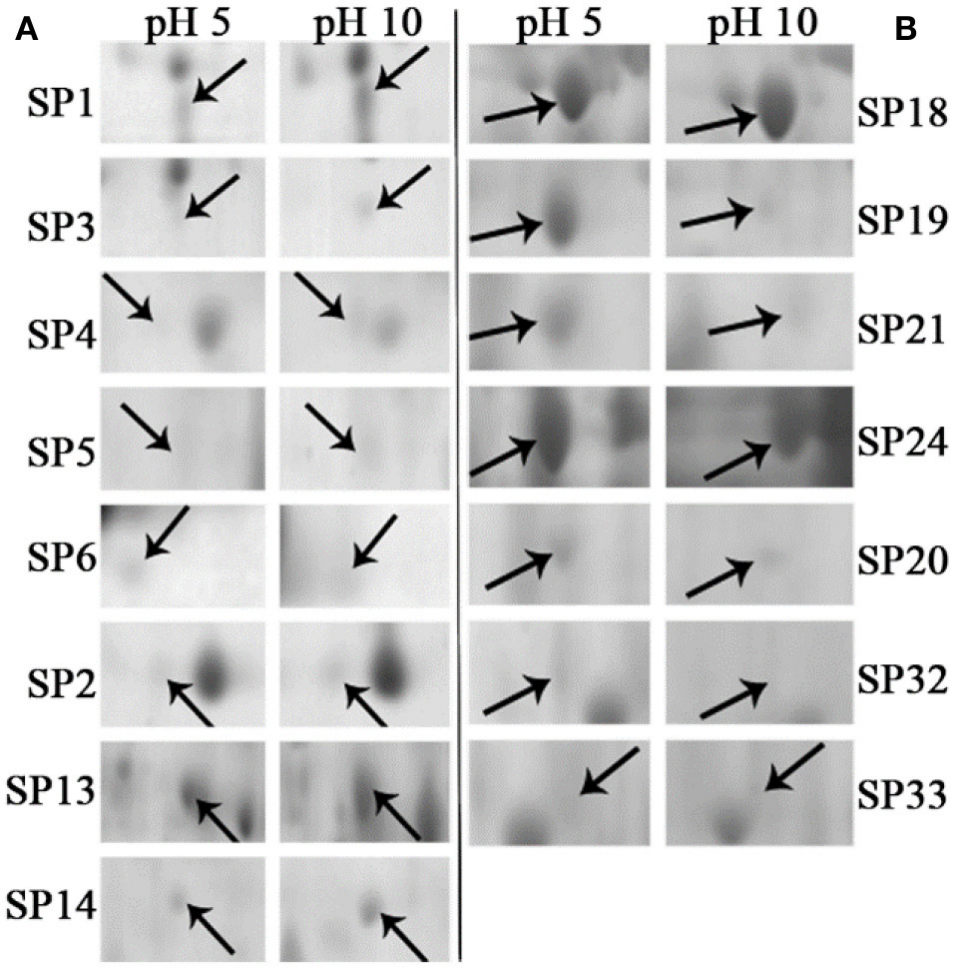
Close-up views of some significant differentially expressed proteins. Some typical spots with significantly differential accumulation patterns were pointed by arrows. **(A)** Up-regulated secretory proteins in *F. proliferatum* under alkaline environment. **(B)** Down-regulated secretory proteins in *F. proliferatum* under alkaline environment. Detail information of proteins were shown in Tables 1, 2.

Total of 17 protein spots were up-regulated at pH 10 compared to pH 5. Most of proteins were predicted as extracellular location by the results of SingalP or SecretomeP. Only spots SP10 and 11, predicted to “regulation of biological process,” were located intracellular. Different from that at pH 5, functions of most differently expressed proteins at pH 10 were mainly involved in oxidation-reduction process, including two thioredoxin reductases (SP1 and 2) and four superoxide dismutases (SOD, SP3-6). In addition, two iron ABC transporter substrate-binding proteins (SP13 and 14) were also up-regulated in the secretome of pH 10-cultured sample. A close-up view of the changes in abundance of these spots was shown in Figure 5A.

### Down-regulated proteins at pH 10

Compared with proteins at pH 5, 22 spots were down-regulated at pH 10 (Table 2). Most of them have the putative functions of carbohydrate metabolism. Interestingly, four of them were involved in the cell wall degrading enzymes (CWDEs), including 1 3-beta-glucanosyltransferase (SP18), related to SPR1-exo-1,3-beta-glucanase (SP19), related to glucan 1 3-beta-glucosidase (SP20) and related to endo alpha-1,4 polygalactosaminidase precusor (SP21). Gluconolactonase (SP23) and probable cellulose (SP24) were also down-regulated at pH 10 compared to pH 5. All of them might play a vital role in the infection of fungi at pH 5. A close-up view of the changes in abundance of these spots was shown in Figure 5B. Three spots related to proteolysis and two spots related to response to stress were also down-regulated. Additionally, three proteins related to nitrogen compound metabolic process (Spots SP26-28) were down-regulated at pH 10, such as serine/threonine protein kinase. All spots were predicted as extracellular proteins according to the positive results from SingalP or SecretomeP except enolase (SP22).

### Transcriptional expression of related genes at pH 5 and 10

**Figure 6:**
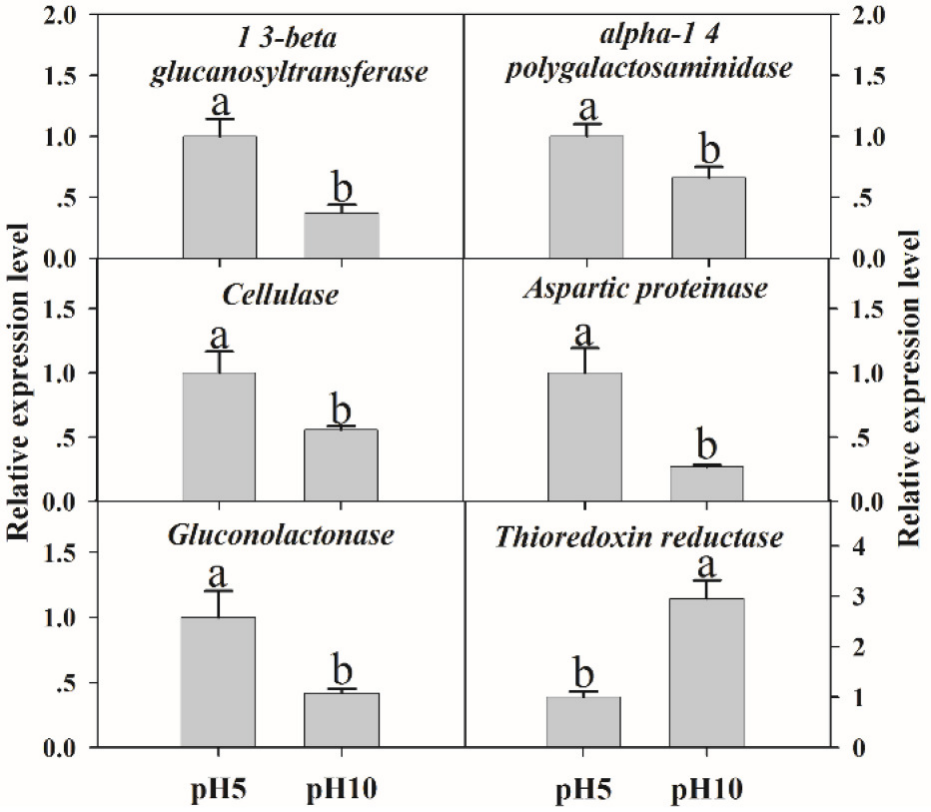
The expression levels of selected genes. The relative expression levels of selected genes were analyzed using qRT-PCR. Each data point represents a mean ± standard error (*n* = 3) and the values with different letters are significantly different (*p* < 0.05).

To verify the validity of proteomic data, we analyzed the expression level of related genes using qRT-PCR. A total of six genes were performed, which were related to cell wall degradation, proteolysis and redox reaction, including *1,3-beta glucanosyltransferase, alpha-1,4 polygalactosaminidase, cellulase, aspartic proteinase, gluconolactonase*, and *thioredoxin reductase* (Figure 6). Result showed that all genes corresponding to CWDEs and proteolysis were down-regulated at pH 10 compared to pH 5 (Figure 6). On the contrary, redox related gene was up-regulated significantly at pH 10 compared to pH 5 (Figure 6). All those genes expression were correlated well with proteomic data. It suggested that the proteomic data was accurate and credible.

## Discussion

### The secreted proteins in *F. proliferatum*

Classical secretory pathway is the most important mechanism to translocate proteins to externally cells with a signal peptide in eukaryotes while proteins without signal peptide can also be secreted out of cells by non-classical secretory pathway (Nickel and Rabouille, 2009). Among these 39 identified proteins, 36 proteins were predicted as the secreted proteins including 26 proteins in classical secretory pathways and 10 proteins in non-classical secretory pathways. Although enolase (SP22), elongation factor (SP10) and 30 S ribosomal protein S13 (SP11) were predicted with negative results from both SignalP and SecretomeP, they were previously reported as secreted proteins in other research (Hughes et al., 2002; Paper et al., 2007). Moreover, enolase might act as virulence factors and be involved in a variety of extracellular functions (Oliveira et al., 2013).

### Alkaline environment induced proteins in *F. proliferatum*

Most up-regulated proteins at pH 10 were involved with oxidation-reduction process, such as thioredoxin reductase and superoxide dismutase. The critical roles of reactive oxygen species (ROS) in many plant pathogen interactions have been well-established (Williams et al., 2011). For pathogens, in response to oxidative stress generated by plant, antioxidant defenses were activated, such as up-regulated SOD (Xu and Chen, 2013). Moreover, SOD was reported to enhance virulence in phytopathogenic fungi (Xie et al., 2010). Deletion or mutation of the *SsSOD1* gene significantly impairs virulence of *S. sclerotiorum* (Xu and Chen, 2013). In the present study, four *SOD*s were up-regulated significantly at pH 10. These results gave a hint that under high pH condition, up-regulation of SOD could not only enhance the defense ability of *F. proliferatum* against ROS stress but also increase the toxicity of fumonisin during infection process. The mycelium proteomics analysis of *F. proliferatum* also proved that pH 10 induced SOD accumulation (Li et al., 2017), which further confirmed the role of SOD in antioxidant defense.

Thioredoxin reductase is another important protein in the antioxidant defense system of fungi, and it is also important to the virulence of *Cryptococcus neoformans* (Missall and Lodge, 2005). Thioredoxin was thought to be important virulence factor induced during pathogens infection or might protect *Phytophthora* from plant counter defenses (Meijer et al., 2014). Moreover, it reported that thioredoxin reductase deletion strain of *Magnaporthe oryzae* was significantly reduced in conidiation and unable to produce expanded necrotic lesions on the leaf surface (Fernandez and Wilson, 2014). In this study, two thioredoxin reductases (Spots SP1 and 2) were significantly up-regulated at pH 10 compared to pH 5 (Table 1), which indicated that thioredoxin reductase might play a vital role in *F. proliferatum* infection process. Interestingly, *thioredoxin reductase* was also induced at pH 10 in gene expression of *F. proliferatum*, which further confirmed our inference.

Porin has been reported to have the function of adhering to host cells (Goo et al., 2006) and pathogen/symbiont recognition (Nyholm et al., 2009). Additionally, the mitochondrial porin was related to the function of SOD, which act as an important pathogenicity/virulence factor for fungi (Budzinska et al., 2007). Recently, it is reported that porin might act as virulence factors modulating host mitochondrial physiology for bacterial survival and immune evasion inside the host cells (Rana et al., 2015). In the present study, porin (Spot SP12) was up-regulated at pH 10 compared with pH 5 (Figure 5A, Table 1), which might also play a crucial role in fungal pathogens.

In this study, two iron ABC transporter substrate-binding proteins (SP13 and 14), were also up-regulated in the secretome of *F. proliferatum* at pH 10. ABC transporter substrate-binding proteins have been reported to participate in nutrient import and protection from stress (Vigonsky et al., 2013). It is reported that exclusive expression of ABC transporters genes is a basic fungal defense reaction when *F. graminearum* was growing on the living plant (Boedi et al., 2016). Our previous study also indicated that in response to BHA treatment, *F. proliferatum* could assemble different ABC transporter substrate-binding proteins to accelerate the nutrient uptake (Li et al., 2016). Collectively, in response to the stress caused by high pH, *F. proliferatum* might assemble different ABC transporter substrate-binding proteins to accelerate the nutrient uptake for the maintaining of growth.

In plants, the alkaline condition results in oxidative burst and alkalization is an essential factor in the induction of defense response (Wojtaszek et al., 1995; Clarke et al., 2005). Tomato fruit apoplastic alkalization activated oxidative burst and SA mediated defense response (Alkan et al., 2012). All these responses of plant under alkaline condition could greatly affect fungal pathogenicity. In response, the fungi adjusted the extracellular proteins in order to survive in the host. Therefore, the high accumulation of these antioxidant enzymes might contribute greatly to the normal growth and pathogenicity of fungus. These antioxidant enzymes involved in the virulence of fungal pathogens may serve as excellent targets for antifungal therapy (Missall and Lodge, 2005). On the other hand, fumonisin content was significantly increased at pH 10 compared to pH 5 (Li et al., 2017), which effectively enhanced the infection ability of *F. proliferatum* and greatly recovered the negative effect of pathogen pathogenicity inhibited by host (Figure 2).

### Alkaline environment inhibited proteins in *F. proliferatum*

It is well known that extracellular proteins related to CWDEs or proteolysis are important for the pathogenicity of plant pathogens. The plant cell wall is an internal physical defensive barrier, and pathogens use extracellular enzymes to degrade the cell wall and invade host (Lakshman et al., 2016). Many researches have reported that CWDEs are involved in the direct degradation of plant tissue, and they have been suggested to be pathogenicity factors of several pathogens, such as pectate lyase (PL), polygalacturonase (PG), etc. (Zhang et al., 2014; Lakshman et al., 2016). In this study, alkaline environment significantly inhibited the accumulation of CWDEs (Table 2), including 1, 3-beta-glucanosyltransferase (SP18), SPR1-exo-1, 3-beta-glucanase (SP19), glucan 1, 3-beta-glucosidase (SP20), endo alpha-1, 4 polygalactosaminidase precusor (SP21), gluconolactonase (SP23), and probable cellulose (SP24). Those results were also reported in *Thielavia reesei* (Adav et al., 2011). On the other hand, qRT-PCR results demonstrated that these proteins under different ambient pH values were also down-regulated at transcriptional level (Figure 6). Therefore, the lower accumulation of these CWDEs under pH 10 condition might result in the slower infection of *F. proliferatum* on banana fruit (Figure 2).

1, 3-beta-glucanosyltransferase was essential for *Fusarium oxysporum* to infect tomato plants (Caracuel et al., 2005). For SPR1-exo-1,3-beta-glucanase and glucan 1, 3-beta-glucosidase, the degradation of β-1,3-glucans may contribute to activating the induction of the programmed cell death in plant cells via generating elicitors in the form of β-(1,3)(1,6)-oligomers (Espino et al., 2010). Gluconolactonase may regulate oxidative stress tolerance and fitness of microorganism (Tarighi et al., 2011). The downregulation of those enzymes might contribute to decrease the infection and growth metabolism of *F. proliferatum* at pH 10. Therefore, our proteomic data indicated that these secreted proteins might have close connection to the biology of *F. proliferatum* during the interaction with its host especially under pH 5 condition.

Besides CWDEs, the proteinase has been also suggested to be involved in plant-pathogen interactions in many studies (Li et al., 2012). It was reported that proteolytic enzymes including aspartic proteases could contribute to the degradation of the host tissue for nutritional acquisition and invasion (Dagenais and Keller, 2009). In the present study, the down-regulation of serine-type protease and aspartic proteinase might contribute to harder infection of *F. proliferatum* at pH 10. Similarly, aspartic proteinase was also down-regulated at transcriptional level (Figure 6). Cell surface enolase of different pathogenic microorganisms could participate in the tissue invasion process and mediate degradation of host tissues and immune evasion (Avilán et al., 2011). The down-regulation of enolase at pH 10 might inhibit the adhesion and invasion of *F. proliferatum* to host tissues then decrease the infection ability of *F. proliferatum*. Enolase was also identified with downregulation in the mycelium proteomics (Li et al., 2017). Therefore, different pH can also cause the significant changes of mycelium proteomics, which might affect the pathogenicity of *F. proliferatum*.

Another protein in the group of “response to stress” is catalase-peroxidase (SP30), which plays a role in defense to oxidative stress. Similar to the upregulation of thioredoxin reductase at pH 10, the upregulation of catalase-peroxidase at pH 5 might also contribute to the normal growth of *F. proliferatum* in response to oxidative stress.

It was worthy to note that the expressions of protein related to nitrogen compound metabolic process (SP26, 27, and 28) were up-regulated under pH 5 condition. Of these proteins, serine/threonine protein kinase with positive results from SecretomeP attracted our attention. Manandhar et al. (2012) reported that the serine/threonine protein kinase could regulate the fusion at the lysosomal vacuole and maintained the fusion/fission dynamics of *Saccharomyces cerevisiae*. Moreover, mitogen-activated protein (MAP) kinases, one of the important type of serine/threonine protein kinases, was reported to be in involved in multiple developmental processes related to sexual reproduction, plant infection and cell wall integrity of *F. graminearum* (Hou et al., 2002). Moreover, the virulence of *F. graminearum* was severely reduced in the *MAPK* mutants (Hou et al., 2002). Our previous study also indicated that serine/threonine protein kinases were greatly reduced by the BHA treatment which might disturbed trafficking and organelle biogenesis beyond the vacuole of *F. proliferatum* (Li et al., 2016). In this study, we observed that serine/threonine protein kinase (SP28) was up-regulated under pH 5 (Table 1), which consequently might contribute to the pathogenic ability of *F. proliferatum*.

## Conclusions

In the present study, *F. proliferatum* cultured under initial pH 5 and 10 conditions both exhibited infection ability on banana fruit. However, pH 10 condition decreased the pathogenicity of the fungus, compared to pH 5. The effect of different pH values on the secretome of *F. proliferatum* was analyzed based on 2-DE. The proteomic data indicated that the secretome of *F. proliferatum* had distinct differences between pH 5 and 10 conditions. Under weak alkaline condition, a great number of CWDEs were down-regulated in *F. proliferatum*, which suggested that the pathogenicity might be significantly inhibited at pH 10 by the inability to degrade the host cell wall effectively. However, a larger number of antioxidant enzymes were up-regulated at pH 10 compared to pH 5, which might contribute greatly to recover the normal growth and pathogenicity of the fungus. We carefully concluded that under pH 5 condition, the *F. proliferatum* secreted more CWDEs for proteolysis, which are more urgently required to degrade the stiffer cell wall of the banana peel. In all, the present study provided a new clue to reveal the reason why banana is susceptible infected by *Fusarium* when pH is below 5.5. It is suggested that *F. proliferatum* is capable of adapting itself with different pH conditions by changing a set of extracellular proteins that prepares itself for encountering stress and infection of the host plant. Further research *in vitro* and in planta is still needed to confirm the exact role of these proteins involved in the infection mechanisms of *F. proliferatum*.

## Author contributions

TL and ZY conceived and designed the study. TL, QW, YW, AJ, HQ, LG, and XD performed the experiments and analyzed the data. TL, ZY, HZ, and YJ drafted and revised the manuscript. All authors participated in the interpretation of data of the manuscript. All authors approved the submission and publication for all aspects of the work.

### Conflict of interest statement

The authors declare that the research was conducted in the absence of any commercial or financial relationships that could be construed as a potential conflict of interest.
