# Supplementary material for: Application of Proteomics for the Investigation of the Effect of Initial pH on Pathogenic Mechanisms of Fusarium proliferatum on Banana Fruit
Source: Front Microbiol. 2017 Nov 29;8:2327. doi: 10.3389/fmicb.2017.02327 (PMC5715366; doi:10.3389/fmicb.2017.02327)
Supplement: Supplementary file 1 [file Table1.DOCX]

**Supplementary Table S1.** Primers used for qRT-PCR in this study.

| **Description** | **Sequence of primer (5’ to 3’)** |
| --- | --- |
| *1 3-beta-glucanosyltransferase* | For: CAAGTCCGAGTCTGTCTGGCAAGTC |
|  | Rev: GGCACCGCTATCATCGTCATCAGAG |
| *Thioredoxin reductase* | For: GACGTTACAGGTCGATGTTGGAAGG |
|  | Rev: CACCGCTGCCTCAATAGAACTCG |
| *endo alpha-1 4 polygalactosaminidase* | For: AGGAAGCGAACGGAGACGAGTAGAA |
|  | Rev: AAGCAGACAGTGATGAAGCCAACCA |
| *Gluconolactonase* | For: GCAACCTCTCTTCTCCCGCTTCTG |
|  | Rev: CACCATCACCGCAGCCAGCATAA |
| *Cellulase* | For: CAGTACCTCGACTCCGACTCCTCTG |
|  | Rev: TCCGCCAGCAGTCTCACCAAGA |
| *Aspartic proteinase* | For: CTTCACAGCACTACCGCCTCAACAA |
|  | Rev: ATTGCCGTCCAGTTCATGCCGATAG |
| *Histone H3* | For: ACTAAGCAGACCGCCCGCAGG |
|  | Rev: GCGGGCGAGCTGGATGTCCTT |
